# Phosphorylation Modulates Ameloblastin Self-assembly and Ca^*2+*^ Binding

**DOI:** 10.3389/fphys.2017.00531

**Published:** 2017-07-27

**Authors:** Øystein Stakkestad, Ståle P. Lyngstadaas, Bernd Thiede, Jiri Vondrasek, Bjørn S. Skålhegg, Janne E. Reseland

**Affiliations:** ^1^Department of Biomaterials, Institute of Clinical Dentistry, University of Oslo Oslo, Norway; ^2^Section for Biochemistry and Molecular Biology, Department of Biosciences, University of Oslo Oslo, Norway; ^3^Department of Bioinformatics, Institute of Organic Chemistry and Biochemistry, Czech Academy of Sciences Prague, Czechia; ^4^Division of Molecular Nutrition, Department of Nutrition, University of Oslo Oslo, Norway

**Keywords:** ameloblastin, phosphorylation, self-assembly, Ca^2+^- binding, enamel, intrinsically disordered proteins, casein kinase 2, protein kinase A

## Abstract

Ameloblastin (AMBN), an important component of the self-assembled enamel extra cellular matrix, contains several *in silico* predicted phosphorylation sites. However, to what extent these sites actually are phosphorylated and the possible effects of such post-translational modifications are still largely unknown. Here we report on *in vitro* experiments aimed at investigating what sites in AMBN are phosphorylated by casein kinase 2 (CK2) and protein kinase A (PKA) and the impact such phosphorylation has on self-assembly and calcium binding. All predicted sites in AMBN can be phosphorylated by CK2 and/or PKA. The experiments show that phosphorylation, especially in the exon 5 derived part of the molecule, is inversely correlated with AMBN self-assembly. These results support earlier findings suggesting that AMBN self-assembly is mostly dependent on the exon 5 encoded region of the AMBN gene. Phosphorylation was significantly more efficient when the AMBN molecules were in solution and not present as supramolecular assemblies, suggesting that post-translational modification of AMBN must take place before the enamel matrix molecules self-assemble inside the ameloblast cell. Moreover, phosphorylation of exon 5, and the consequent reduction in self-assembly, seem to reduce the calcium binding capacity of AMBN suggesting that post-translational modification of AMBN also can be involved in control of free Ca^2+^ during enamel extra cellular matrix biomineralization. Finally, it is speculated that phosphorylation can provide a functional crossroad for AMBN either to be phosphorylated and act as monomeric signal molecule during early odontogenesis and bone formation, or escape phosphorylation to be subsequently secreted as supramolecular assemblies that partake in enamel matrix structure and mineralization.

## Introduction

Ameloblastin (AMBN) is an enamel extracellular matrix protein (Paine and Snead, 1997; Bartlett et al., 2006; Chun et al., 2010a; Geng et al., 2015) with multiple roles during odontogenesis, including mesenchymal and ectodermal cell differentiation (Fukumoto et al., 2004, 2005; Iizuka et al., 2011; Kitagawa et al., 2011) and enamel and dentin biomineralization (Nanci et al., 1998; Nakamura et al., 2006). Formation of enamel has been shown to be dependent on self-assembly of enamel matrix proteins such as AMBN (Wazen et al., 2009; Poulter et al., 2014; Lu et al., 2016a,b; Wald et al., 2017), and amelogenin (AMEL; Fincham et al., 1994; Paine et al., 2000; Moradian-Oldak et al., 2002). Self-assembly of AMBN depends mostly on the exon 5 derived region located in the N-terminal half of the molecule (Wald et al., 2013). The N-terminus is itself processed from complete AMBN and it is found within self-assembled fibrillary structures in the sheath space (Geng et al., 2015), that define the boundaries of the enamel prisms. AMBN and AMEL are both elongated intrinsically disordered proteins that are hydrophobic and may also function to concentrate calcium phosphate minerals for hydroxy apatite (HAp) crystal nucleation and growth in enamel. AMBN spontaneously self-assemble (Wald et al., 2013), yet the control of self-assembly remain obscure. AMBN is potentially phosphorylated by protein kinase A (PKA) and casein kinase 2 (CK2) in predicted sites that cover both the N-terminus and C-terminus, including sites in exon 5 and exon 6 (Toyosawa et al., 2000; Lee et al., 2003; Perdigao et al., 2004, 2009).

The enamel sheath is laid down as one unit and embeds the prisms in their entire length from the ameloblast cells to the dentin surface. Full length AMBN self-assembles (Wald et al., 2013) and may initially only populate the enamel sheath. While still a part of unprocessed AMBN, the C-terminus is suggested to be involved in docking the sheath to the ameloblast cell surface through heparin binding domains (Sonoda et al., 2009). Complete AMBN is however not found *in vivo* other than as trace elements (Murakami et al., 1997; Uchida et al., 1998). One may envision that the C-terminus must be processed from the full length AMBN protein to allow for continuous growth of the sheath in the growth direction of the enamel prisms. *In silico* modeling of the full-length folded protein also suggest Ca^2+^ binding in the C-terminus region (Vymetal et al., 2008). The C-terminus processing products are quickly removed from the ECM and are only found in the sheath space in close proximity to the ameloblast cell surface (Uchida et al., 1997; Geng et al., 2015). The specific mechanism(s) for how AMBN C-terminus and its processing products contribute to enamel organization and mineralization and especially sheath formation is still poorly understood.

Transgenic mice that express a version of AMBN C-terminus (Ma et al., 2016) that is phosphorylated in a site close to several predicted Ca^2+^ binding sites (Zhang et al., 2011), show disturbance in enamel mineralization. Binding of Ca^2+^ may regulate crystal nucleation, mineralization and cellular attachment (Zhang et al., 2011). It is therefore important to see if more of the *in silico* predicted phosphorylation sites actually are phosphorylated and thus can partake in the regulation of AMBN function(s). It is also interesting to see if specific phosphorylation sites directly influence self-assembly and/or calcium binding in the full-length molecule and the major processing products. Finally, to better understand the relation between phosphorylation, Ca^2+^ binding and molecular organization, it is necessary to analyze whether Ca^2+^ binding itself has effect on the structural order in self-assembled and non-assembled AMBN.

## Materials and methods

### Radioactively labeled kinase assay

Purified AMBN DelEx5 was incubated with 1,000 units of recombinant PKA Cα1 (New England Biolabs, Ipswich, MA) in PKA reaction buffer, or 5,000 units CK2 (New England Biolabs) in CK2 reaction buffer supplied with 0.1 μl γ-[32P] ATP (~6,000 Ci/mmol, PerkinElmer, Waltham, MA, USA) in a total volume of 20 μl for 30 min at 30°C. The reactions were terminated by addition of 4 μl SDS loading buffer and boiled for 10 min. Subsequently the samples were separated by SDS-PAGE in 12% Tris-HCl (BioRad, Hercules, CA, USA), and the bands were exposed by Coomassie stain and the radioactivity was detected by exposure to CL-Xposure film (ThermoFischer Scientific, Waltham, MA, USA).

### Kinase assay

His-tagged (1 μg) PKA Cα1 (Millipore, Billerica, MA, USA,) or his-tagged (1 μg) CK2 (ATGen Ltd, Bundang-gu, Seongnam-si, Gyeonggi-do, South Korea,) was incubated with purified AMBN-WT (100 pmol), C-terminus (314 pmol), N-terminus (100 pmol), or DelEx5 (100 pmol) in CK2 or PKA reaction buffer supplied with 1 mM ATP in a total volume of 20 μl at 30°C for 24 h. Ten picomoles from each of the samples were submitted to LC-ESI-MS analyses. The remaining of the samples were boiled in Laemmli buffer, loaded on a 12% Ready Gel Tris-HCL (BioRad), and separated by electrophoresis, and the gel stained for phospho-proteins with Pro-Q® Diamond Phosphoprotein Gel Stain and destained with Pro-Q® Diamond Phosphoprotein Gel Destaining Solution (ThermoFisher Scientific, Waltham, MA, USA) according to manufacturer's protocol. The phosphor luminiscent gel bands were then scanned at in ChemiDoc XRS+ imaging system (BioRad) at 510 nm.

### Liquid chromatography electrospray ionization-mass spectrometry (LC-ESI-MS)

To detect phosphorylation sites of AMBN, 10 pmol of protein was digested by adding 0.2 μg trypsin in 20 μl 25 mM ammonium bicarbonate and incubation for 16 h at 37°C. The digestion was stopped by adding 2 μl 5% formic acid. The generated peptides were purified using an OMIX C18 (Agilent, Santa Clara, CA, USA), and dried using a Speed Vac concentrator (Concentrator Plus, Eppendorf, Hamburg, Germany).

The tryptic peptides were dissolved in 10 μl 0.1% formic acid/2% acetonitrile and 5 μl analyzed using an Ultimate 3000 RSLCnano-UHPLC system connected to a Q Exactive mass spectrometer (ThermoFisher Scientific) equipped with a nano electrospray ion source. For liquid chromatography separation, an Acclaim PepMap 100 column (C18, 2 μm beads, 100 Å, 75 μm inner diameter, 50 cm length) (Dionex, Sunnyvale CA, USA) was used. A flow rate of 300 nL/min was employed with a solvent gradient of 4–35% B in 47 min, to 50% B in 10 min and then to 80% B in 3 min. Solvent A was 0.1% formic acid and solvent B was 0.1% formic acid/90% acetonitrile. The mass spectrometer was operated in the data-dependent mode to automatically switch between MS and MS/MS acquisition. Survey full scan MS spectra (from m/z 300 to 2,000) were acquired with the resolution R = 70,000 at m/z 200, after accumulation to a target of 1e6. The maximum allowed ion accumulation times were 60 ms. The method used allowed sequential isolation of up to the 10 most intense ions, depending on signal intensity (intensity threshold 1.7e4), for fragmentation using higher-energy collisional induced dissociation (HCD) at a target value of 10,000 charges and a resolution R = 17,500 Target ions already selected for MS/MS were dynamically excluded for 60 s. The isolation window was m/z = 2 without offset. For accurate mass measurements, the lock mass option was enabled in MS mode.

Data were acquired using Xcalibur v2.5.5 and raw files were processed to generate peak list in Mascot generic format (^*^.mgf) using ProteoWizard release version 3.0.331. Database searches were performed using Mascot in-house version 2.4.0 to search the SwissProt database (Human, 20.279 proteins), assuming the digestion enzyme trypsin at maximum one cleavage site, fragment ion mass tolerance of 0.05 Da, parent ion tolerance of 10 ppm, and oxidation of methionines, and acetylation of the protein N-terminus as variable modifications. Scaffold (version Scaffold_4.4.8, Proteome Software Inc., Portland, OR) was used to validate MS/MS based peptide and protein identifications. Peptide identifications were accepted if they could be established at >95.0% probability by the Scaffold Local FDR algorithm.

### Dynamic light scattering (DLS)

In dynamic light scattering (DLS), a particle in solution is illuminated by laser and scatter light that fluctuate with the random movement (Brownian motion) of the particles. As movement of a particle is inversely correlated with its mass, the light scattering fluctuations may be used to estimate particle size. DLS was measured in a Zetasizer Nano ZS (Malvern Instruments Ltd, Worcestershire, UK) in DTS1070 disposable foldable capillary cells (Malvern Instruments Ltd) at 12°C, using a backscatter angle of 173°, an equilibration time of 5 min and three replications per sample. Analysis was done in the Zetasizer Software v7.11 (Malvern Instruments Ltd). The dispersant was chosen in the software as water with a refractive index of 1.330 and viscosity 0.8872 cP, the particle protein with refractive index 1.450 and absorption 0.001. The distribution analysis was done using the protein analysis option in the software which is a non-negative least squares analysis with an automatically determined regularizer and L-curve analysis.

### Small angle X-ray scattering (SAXS)

Small angle X-ray scattering is used to gather information about the structure of proteins in solution. This information is obtained by analyzing the intensity of photons (I) scattered in terms of the scattering vectors (s). To analyze the degree of folding of macromolecules, I(s)·(s)^2^ against (s nm^−1^) is plotted in a Kratky plot (Putnam et al., 2007). Folded macromolecules have a high I at lower values of (s), illustrated as a peak in the plot. For a disordered and elongated protein, the function of I is linear and reaches a plateau at higher (s) values. SAXS was performed at the European Synchrotron Radiation Facility (ESRF) in Grenoble at the BioSAXS beamline BM29 using the automated sample changer in flow mode at 20°C, with 10 frames measured, of 1 second duration, per injected sample and an injected volume of 50 μl. The quartz capillary sample cell diameter was 1.8 mm, detector distance 2.867 m, with wavelength λ = 1.008 Å. A buffer baseline was measured before and after each measurement using 50 μl buffer. Before each measurement the protein was diluted into concentrations ranging from 0.2 to 1.8 mg/ml. The size of the particles that is analyzed is limited by the resolution of the instrument. As is the case for the beamline intended for biological SAXS at ESRF, the largest object that can be resolved is 50 nm in size (D_max_); thus all particles larger than that will be presented with a size similar to D_max_ of 50 nm. The average size of the particle is presented as radius of gyration (R_g_). Molecular weight of the protein in solution is calculated based the known concentration of protein and the volume calculated. A detailed description of the calculation method can be found at: http://www.esrf.eu/home/UsersAndScience/Experiments/MX/About_our_beamlines/bm29/computing-environment.html.

### Experimental design

**Figure 1:**
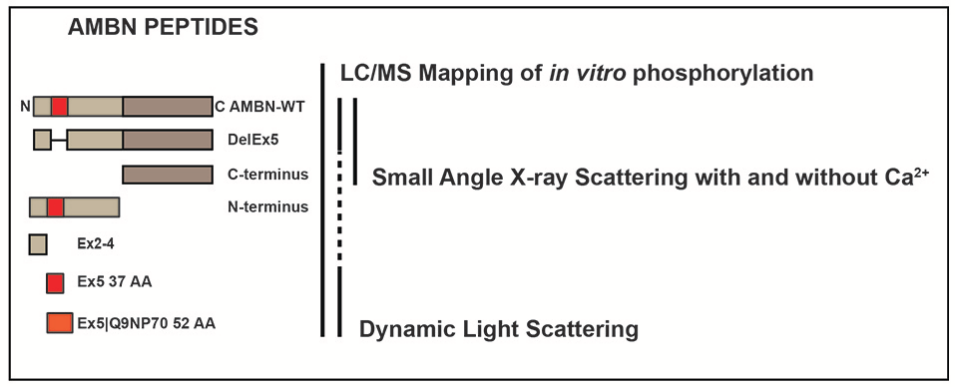
The experimental design indicating the various recombinant AMBN proteins and peptides and test methods used in this study. Recombinant human AMBN-WT, N-terminus, C-terminus, DelEx5, Ex2-4, Ex5 proteins, and peptides were tested with LC-ESI-MS mapping of all peptides phosphorylated *in vitro*, dynamic light scattering (DLS) of Ex5, Ex5|Q9NP70, AMBN-WT, and DelEx5 and small angle x-ray scattering (SAXS) of AMBN-WT, DelEx5, and C-terminus with and without Ca^2+^.

An outline of the experimental design is presented in Figure 1, indicating the various recombinant AMBN proteins and peptides and test methods used in this study.

The procedure for cloning, expression and purification has previously been described by Wald et al. (2013). The various constructs were wild type, full-length AMBN (AMBN-WT), a variant deleted for exon 5 (DelEx5), a C terminal fragment representing amino acids 223–447 (C-terminus), a N-terminal fragment representing amino acid 27–222 (N-terminus). In addition, peptide representing exon 2–4 (Ex2–4 34 AA, VPFFPQQSGTPGMASLSLETMRQLGSLQRLNTLS), 37 amino acids of exon 5 (Ex5 37 AA, YSRYGFGKSFNSLWMHGLLPPHSSLPWMRPREHETQQ) and Ex5 including the splice variant of exon 6 designated Ex5|Q9NP70 (52 AA, YSRYGFGKSFNSLWMHGLLPPHSSLPWMRPREHETQQYEYSLPVHPPPLPSQ) were included for *in vitro* phosphorylation in addition to AMBN-WT, DelEx5, N-terminus and C-terminus. All the peptides were in a buffer containing 20 mM Tris-HCl, and 50 mM NaCl. The concentration of the various AMBN proteins and peptides were quantitated using the standard colorimetric Bradford protein assay (Bradford, 1976).

For the DLS experiments the peptide Ex5 37 AA, Ex5|Q9NP70 52 AA and full-length AMBN (AMBN-WT) were employed. Finally, the SAXS experiments were conducted on AMBN-WT, DelEx5, C-terminus. The latter experiments were performed in the presence and absence of Ca^2+^ (0.01 mM).

## Results

**Figure 2:**
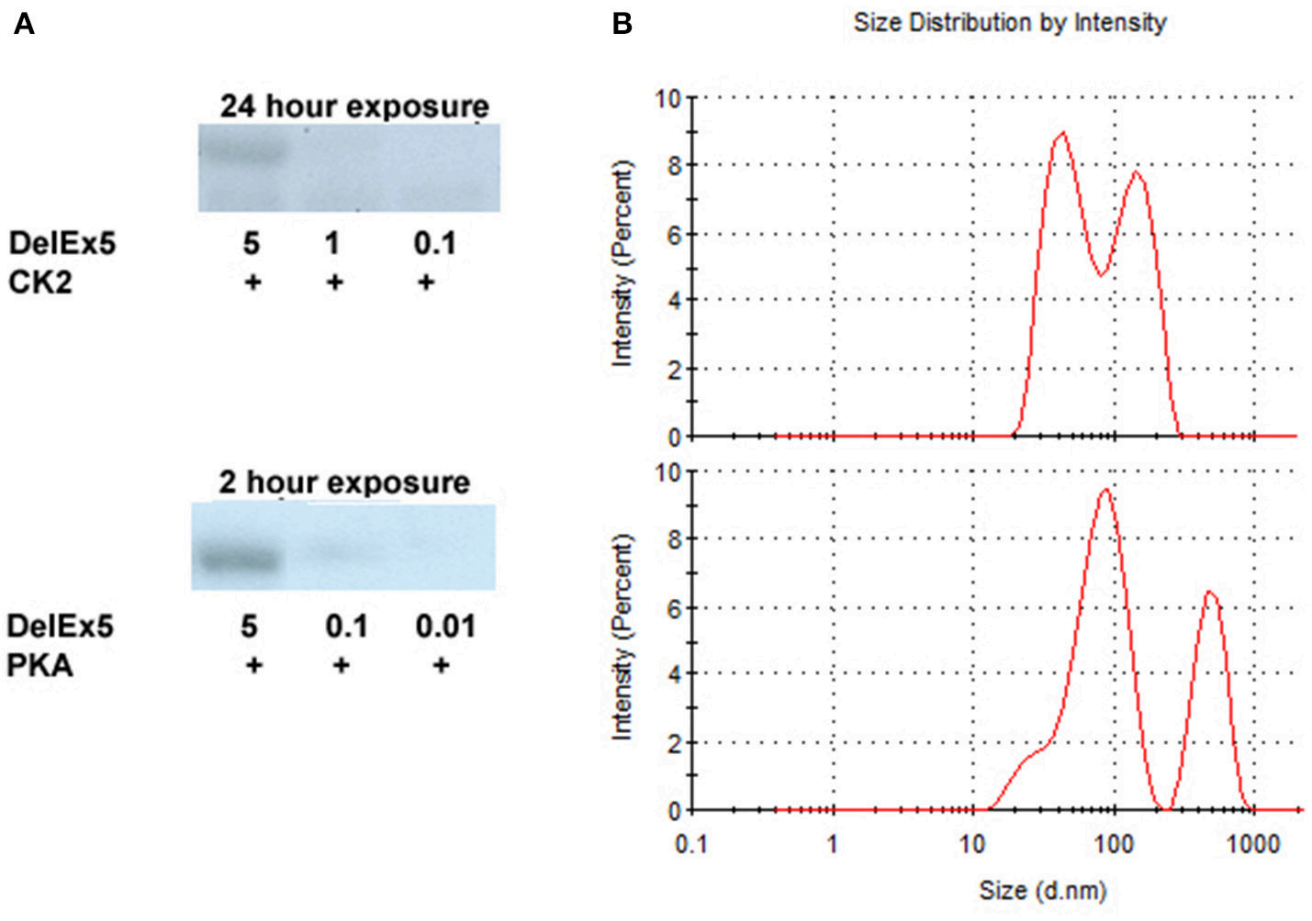
Autoradiography of DelEx5 (0.01–5 μg) phosphorylated with CK2 and PKA **(A)**. Dynamic light scattering of AMBN-WT (upper panel) and AMBN-WT incubated with 5% glycerol (lower panel) **(B)**. In these experiments the phosphorylation of DelEx5 by CK2 required an exposure time of 24 h to match a 2 h exposure of DelEx5 phosphorylated by PKA.

Both PKA and CK2 phosphorylated AMBN DelEx5 in a dose–dependent fashion (Figure 2A). It should be noted that phosphorylation appeared more pronounced for PKA compared to CK2 as the exposure time was 2 h for PKA and 24 h for CK2 using 5 μg recombinant AMBN DelEx5. Glycerol, which is known to dissociate the tight structure between proteins (Contaxis and Reithel, 1971) were added in the range 5–30% to AMBN protein solutions to assess its effect on the size distribution of AMBN complexes. Under the present conditions, a concentration of 5% glycerol caused the AMBN complexes to dissolve into monomers (Figure 2B). Consequently, 5% glycerol was included in the reaction solutions to ensure that molecules did not self-assemble during the kinase assays.

### Differential phosphorylation of AMBN by PKA and CK2

**Figure 3:**
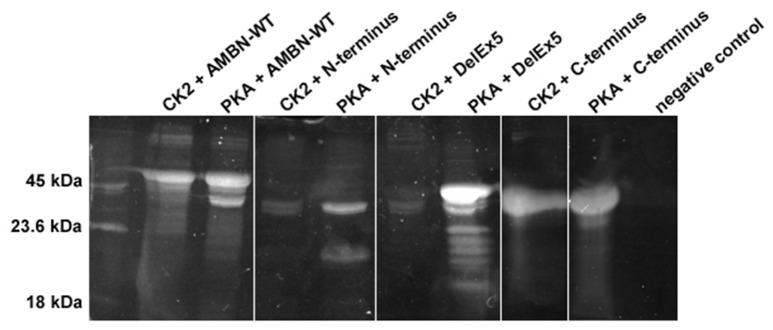
Composite image representative of phosphorylated AMBN proteins and peptide by casein kinase2 (CK2) and protein kinase A (PKA). C-terminus, and AMBN-WT, N-terminus, and DelEx5 was phosphorylated by CK2 or PKA for 24 h, separated in 12% SDS-PAGE, and stained by ProQ diamond phosphorous stain. The intensity of bands between lanes can be interpreted as a semi-quantitative measurement of phosphorylation.

CK2 phosphorylated AMBN-WT and the C-terminus fragment effectively because dominant bands were observed by SDS-PAGE and phosphoprotein staining. However, neither the AMBN molecule without exon 5 (DelEx5) nor the N-terminus peptide could be effectively phosphorylated with CK2 (Figure 3). PKA phosphorylated both the N-terminus and the C-terminus fragments effectively. On the other hand, PKA was less effective in phosphorylating AMBN-WT and the DelEx5 fragment as visible by the presence of additional significant bands with different molecular weights (Figure 3).

### Differential phosphorylation of regions in self-assembled AMBN and peptides encoded by the exons 2–6

**Figure 4:**
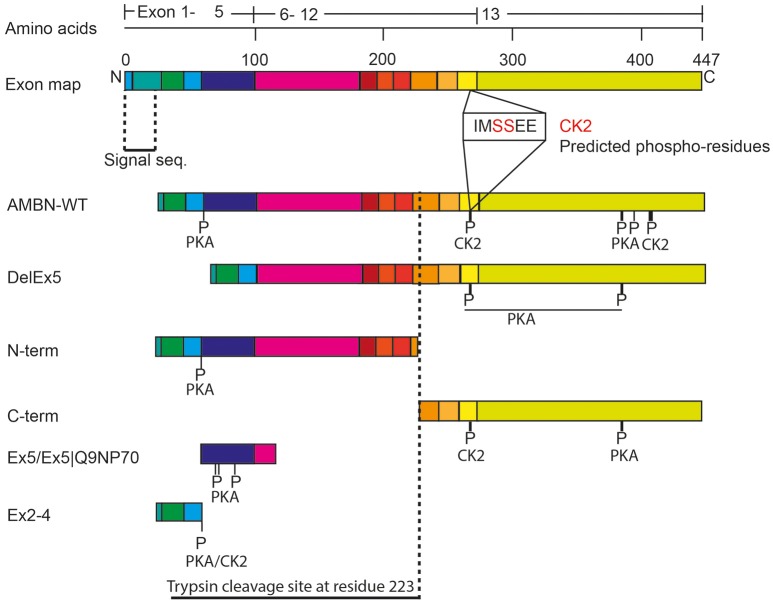
Schematic overview of the phosphorylated sites in AMBN. The exon organization in relation to the various recombinant proteins and peptides are illustrated. P-indicate position of phosphorylated residues. The vertical dotted line indicates the known trypsin cleavage site between A222 and L223 (Iwata et al., 2007).

LC–ESI-MS analysis of aliquots from the experiments conducted in Figure 3, revealed a higher incidence of phosphorylation in the C-terminus region of AMBN-WT than in the self-assembled N-terminus and the N-terminus region in AMBN-WT. Moreover, the C-terminus was phosphorylated in additional sites as a region in the self-assembled AMBN-WT than in the C-terminus monomeric peptide (Figure 4 and Table 1).

**Table 1:** Ratio of phosphorylated residues of AMBN.

		**exon 2–4**	**exon 5–6**		**exon 12**	**exon 13**	
		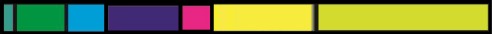					
**Fragments**	**Enzyme**	**T-58**	**S-70,-73,-85**		**S-261/2**	**S-377**	**S-397/8**
AMBN-WT	CK2	0% (0/8)	NF	NF	50% (5/5)	5% (1/21)	75%(3/1)
	PKA	8% (1/11)	0% (0/2)	(0/2)	0% (0/9)	32% (11/23)	0% (0/5)
DelEx5	CK2	N/F		N/F	0% (0/29)	0% (0/46)	N/F
	PKA	N/F		N/F	5% (2/39)	28% (10/26)	N/F
N-terminus	CK2	N/F	NF	N/F			
	PKA	8% (1/11)	0% (0/1)	N/F			
C-terminus	CK2				59% (23/16)	0% (0/3)	N/F
	PKA				0% (0/29)	42% (25/34)	N/F
Ex2–4	CK2	11% (1/8)					
	PKA	71% (10/4)					
Ex5	CK2		N/F				
	PKA		84% (5+9+7/4)				
Ex5|Q9NP70	CK2		0% (0/12)				
	PKA		80% (1+7+4/3)				

*The amino acids phosphorylated are indicated in the upper row (T, threonine; S, serine). The percentage of phosphorylated sites found by LC-ESI-MS column is calculated from the ratio of phosphorylated fragments to number of total identified fragments. The number of phosphorylated and non-phosphorylated residues is given in brackets. N/F, sequence not found*.

DLS analyses of peptides Ex5 and Ex5|Q9NP70 indicated no sign of self-assembly (results not shown) confirming the findings reported by Wald et al. (2013). Kinase assays with CK2 and PKA performed on peptides Ex2–4, Ex5, and Ex5|Q9NP70 were analyzed in LC-ESI-MS indicating efficient phosphorylation by PKA. The exons 2–5 encode most of the N-terminal part of AMBN. Yet, the N-terminus fragment was only partly phosphorylated by PKA; both as a self-assembled fragment, and as a region in the full length AMBN-WT. In overall PKA phosphorylated sites in both the N-terminus and the C-terminus part of both the DelEx5 and the full-length AMBN proteins but with un-equal efficacy. This is in accordance with several molecular weights of phosphorylated proteins separated as multiple bands shown in Figure 3 (lanes 3 and 11, respectively). For a detailed overview of phosphorylation sites and incidence in all peptides see Figure 4 and Table 1.

### The effect of self-assembled AMBN on structure and Ca^2+^ binding

**Figure 5:**
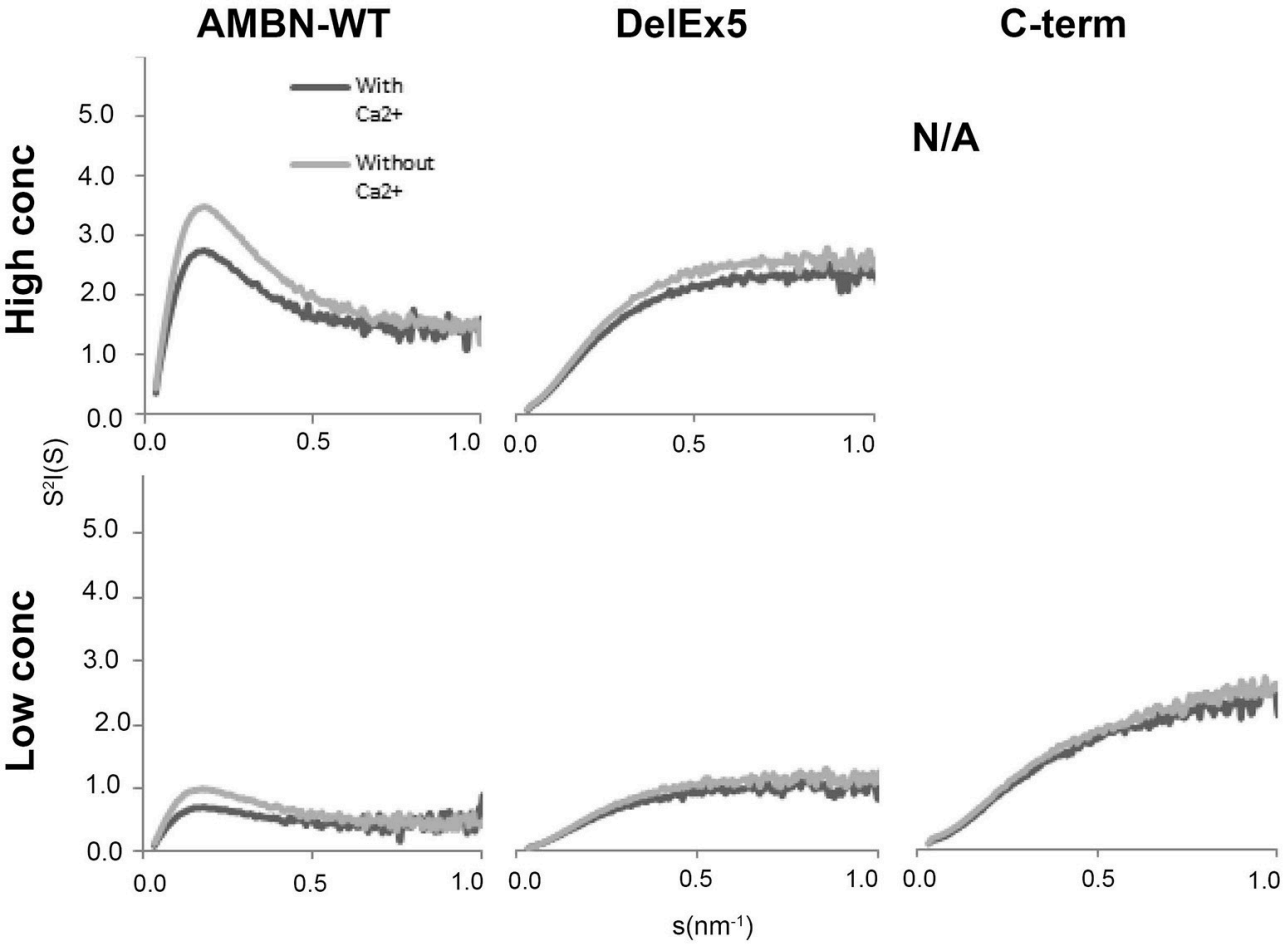
Short Angle X-ray Scattering (SAXS) Kratky plots of AMBN-WT, DelEx5, and C-terminus with or without Ca^2+^. High concentration corresponds to 0.7, 0.88 mg/ml, and N/A, respectively, whereas low concentration corresponds to 0.4, 0.44, and 0.44 mg/ml, respectively. N/A means not analyzed due to lack of material.

Only AMBN-WT displayed a peak that is typical for folded macromolecules. This feature of AMBN-WT was altered in the presence of Ca^2+^ in all concentrations (Figure 5). DelEx5 had a disordered profile comparable to the C-terminus. The disorder in DelEx5 and C-terminus seemed un-affected by exposure to Ca^2+^ (Figure 5).

SAXS analyzes of AMBN-WT indicated structures above 50 nm in size (D_max_) while radius of gyration (R_g_) was calculated to 14.5 nm that was that increased upon exposure to Ca^2+^ in all the measured concentrations of AMBN (Table 2). For the self-assembled AMBN with a size larger than the D_max_ resolution, Rg may here be viewed as the dimensions of AMBN in terms of the width of its fibrils. For DelEx5 and C-terminus within the D_max_ range, both Rg and D_max_ may be viewed as dimension of the individual proteins. For DelEx5 and the C-terminus, the Rg was smaller and less affected by Ca^2+^ compared to AMBN-WT. The default settings were employed in calculating Rg for comparison of disordered proteins with fibrillary AMBN proteins (AMBN-WT).

**Table 2:** Results from small angle X-ray scattering (SAXS) of AMBN, DelEx5 and C-terminus.

**Sample**	**R_g_ (Gu) nm**	**R_g_ (Gn) nm**	**MMVol. kDa**	**D_max_**	**Qual. (%)**
WT 1 mg/ml	14.6	15.1	3,027–4,037	51.1	90.4
WT 0.7 mg/ml	14.6	15.1	2,948–3,931	51.0	90.1
WT 0.4 mg/ml	14.2	14.8	2,908–3,878	49.7	90.8
WT 0.2 mg/ml	14.2	14.9	2,923–3,897	49.7	89.7
WT 0.7 mg/ml + Ca^2+^	14.9	15.3	3,067–4,889	52.0	92.5
WT 0.4 mg/ml + Ca^2+^	14.8	15.2	3,017–4,022	51.7	91.1
WT 0.2 mg/ml + Ca^2+^	14.9	15.5	3,159–4,212	52.2	83.3
Del Ex 5 1.8 mg/ml	5.7	5.9	74–98	19.9	65.6
Del Ex 5 0.9 mg/ml	5.9	6.0	76–102	20.3	65.7
Del Ex 5 0.4 mg/ml	5.7	5.9	72–96	20.0	65.5
Del Ex 5 0.3 mg/ml	5.8	6.0	75–100	20.4	59.3
Del Ex 5 0.9 mg/ml + Ca^2+^	5.7	5.8	71–95	19.1	65.4
Del Ex 5 0.4 mg/ml + Ca^2+^	5.8	5.8	72–96	19.3	59.8
C-terminus 0.4 mg/ml	4.6	4.6	31–42	14.9	64.7
C-terminus 0.3 mg/ml	4.4	4.3	27–36	13.5	64.9
C-terminus 0.4 mg/ml + Ca^2+^	4.8	4.7	32–43	16.2	69.6
C-terminus 0.2 mg/ml + Ca^2+^	4.6	4.8	33–43	15.1	58.1

*The distribution of AMBN in space around its axis is given as gunier (Gu) and Gnome (Gn) radius of gyration, molecular mass is given in kilo Daltons (MMVol. kDa) and the maximum size of particle is given as D_max_, and the quality (Qual) of the data is given in % refers to the autoRg calculation of Rg (Gu). For detail, see http://www.esrf.eu/home/UsersAndScience/Experiments/MX/About_our_beamlines/bm29/computing-environment.html*.

The molecular weight calculated was higher than expected for DelEx5 ranging between 70 and 90 kDa while the C-terminus had close to expected MW ranging between 27 and 43 kDa (Table 2). AMBN-WT was calculated a very high molecular weight, indicative of some sort of supramolecular assembly but these data are restricted by the resolution limit of the instrument at 50 nm (D_max_).

## Discussion and conclusion

AMBN is a calcium-binding phosphoprotein found within the self-assembled enamel extracellular matrix. The results presented suggest that the phosphorous acceptor sites identified here are hidden in self-assembled AMBN and could point at a mechanism for regulation of self-assembly through the cAMP-PKA pathway. During formation of enamel, G-protein coupled receptors that may influence cAMP levels are expressed in differentiated and differentiating ameloblast cells (Bawden et al., 1996). Furthermore, Enamel extracellular Matrix Derivative (EMD) enhance cAMP levels in human epithelial and periodontal ligament cells (PDL; Lyngstadaas et al., 2001). This suggests the cAMP-PKA signaling pathway is likely to coincide with AMBN expression.

Located in the N-terminus, the regions encoded by exon 5 and exon 6 are both found to be important for biomineralization of enamel (Wazen et al., 2009) and bone (Lu et al., 2016a,b). We recently showed that self-assembled AMBN and monomeric peptides encoded by exon 5 influence differentiation of human mesenchymal stem cells (hMSC; Stakkestad et al., 2017). Tamburstuen et al. also showed that AMBN is expressed in hMSC, stromal stem cells, and bone cells (Tamburstuen et al., 2011a), and that upstream regulatory elements essential for osteogenesis, adipogenesis, and chondrogenesis are present directly upstream of the human *AMBN* gene (Tamburstuen et al., 2011b). AMBN is functional, and is also expressed in adult bone repair (Nakamura et al., 2006; Spahr et al., 2006; Tamburstuen et al., 2010). During tooth development AMBN is induced by epithelial-mesenchymal interactions and expressed in MSC and epithelial cells (Fong et al., 1998; Takahashi et al., 2012) and in the pre-secretory and secretory stages of ameloblasts development (Nanci et al., 1998). Exon 5 and exon 6 knock down experiments in mice did however, not have any observable effects on the pre-secretory stages of ameloblasts development (Wazen et al., 2009). As this abrogation of AMBN self-assembly did not cause developmental effects, one may argue that AMBN self-assembly and interaction with AMEL is important only in enamel matrix secretion and mineralization.

From our SAXS results we can infer that AMBN self-assembly mostly rely on the exon 5 derived region as variants lacking this region (DelEx5) were all disordered. Moreover, self-assembly of AMBN seems directly related to biomineralization since only intact, self-assembled AMBN-WT is structurally influenced by Ca^2+^. The here reported findings support previous results (Wazen et al., 2009; Lu et al., 2011, 2016a; Wald et al., 2017) suggesting that the exon 5 deletion variant (DelEx5) cannot bind Ca^2+^efficiently and compromises biomineralization when present in the enamel matrix. This also corresponds well with previous *in silico* analysis of AMBN that suggested the calcium-binding site located to the C-terminus part of the protein (Vymetal et al., 2008; Zhang et al., 2011) and Ca^2+^ binding in the isolated 27 and 29 kDa C-terminal processing products (Yamakoshi et al., 2001). Interestingly the C-terminus protein in solution (monomeric) was inefficient in binding Ca^2+^, confirming the previous observation by (Wald et al., 2011).

We here suggest that AMBN function depends on whether the molecules are self-assembled as a matrix component or exist as a mono-disperse soluble molecule. The observed phosphorylation of the exon 5 encoded region probably modulate the ability for self-assembly. In the phosphorylated, soluble form AMBN probably have functions that are not yet fully elucidated, possibly including roles in cell signaling and stem cell recruitment and differentiation. AMBN was initially predicted to function as a signaling molecule (Cerny et al., 1996; Fukae et al., 2006) during early tooth formation, but also assigned to be involved in generating the prismatic structure of mature enamel (Robinson et al., 1998). This ambiguity of the AMBN molecule may rest on the phosphorylation status of the molecule, providing a “one-molecule-to-many-functions” system that is observed also in other intrinsically disordered proteins in the Osteonectin (aka SPARC) family (Kawasaki et al., 2007). In this way AMBN can both be involved in the self-assembled, insoluble, fibrillary structures in the sheath space (Geng et al., 2015) interacting with the major enamel protein AMEL through the exon 5 derived region (Su et al., 2016), and act in cell-matrix attachment (Zhang et al., 2011), and as a signaling molecule involved in cell signaling and extracellular matrix feedback.

We also suggest that processing of AMBN is involved in transport and release of Ca^2+^ to the enamel extracellular compartment: Ca^2+^ are then brought out of the cell bound to and supported by the self-assembled chelating AMBN complex, thus allowing easy transport against the high osmotic gradient present in the supersaturated enamel extracellular milieu. Despite the numerous predicted phosphor acceptor sites in AMBN (Toyosawa et al., 2000; Lee et al., 2003), few residues of AMBN have been found to be phosphorylated (reviewed in Delsuc et al., 2015). It is possible that phosphorylation of AMBN is more efficient *in vivo*, but is effectively removed by alkaline phosphatases before it can be detected. Especially the CK2 predicted sites in the C-terminus (Toyosawa et al., 2000; Lee et al., 2003), that does not partake in self-assembly, are suggested to be functional during biomineralization (Ma et al., 2016). The C-terminus of AMBN is processed from the self-assembled AMBN full length protein by metalloproteases (Iwata et al., 2007; Chun et al., 2010b) and the 20S proteasome subunit (Geng et al., 2015), and may subsequently release phosphate and Ca^2+^ into the enamel fluid for HAp crystal nucleation and/or growth.

In conclusion, phosphorylation seems to regulate molecular organization and Ca^2+^ binding in AMBN. In a phosphorylated state, the molecules do not organize into supramolecular assemblies and Ca^2+^ binding is probably insignificant. However, when phosphorylation is lacking, the molecules self-assemble into organized structures that can bind Ca^2+^. Calcium is probably secondary in modulation of AMBN organization as un-phosphorylated molecules also form assemblies without it. Thus, calcium alone has only minor effect on the structural order of AMBN. Based on our findings, we speculate that phosphorylation provide a switch for the function of the molecule, either to act as an un-phosphorylated and insoluble, Ca^2+^ binding complex in mineralizing extracellular matrices, or as a phosphorylated and soluble signal molecule in tissue development and repair. Further work needs to be done with respect to the kinetics and quantitation of the extent and effect(s) on phosphorylation, and findings need to be confirmed in *in vivo* models before the complete picture of AMBN functions in hard tissue development, mineralization and repair is revealed.

## Author contribution

ØS: Designed the setup of experiments, performed experiments, and drafted the manuscript. SL: Participated in experimental design and in drafting the manuscript, BT: Performed experiments, participated in experimental design, and drafting the manuscript, JV: Provided material, participated in experimental design, and drafting the manuscript, BS: Participated in experimental design, and drafting the manuscript, JR: Participated in experimental design, and drafting the manuscript.

### Conflict of interest statement

The authors declare that the research was conducted in the absence of any commercial or financial relationships that could be construed as a potential conflict of interest. The reviewer MG and handling Editor declared their shared affiliation, and the handling Editor states that the process met the standards of a fair and objective review.
